# A Misplaced S2 Alar-Iliac Screw Causing L5 Spinal Nerve Injury: A Report of a Rare Case

**DOI:** 10.7759/cureus.73638

**Published:** 2024-11-13

**Authors:** Toru Funayama, Yohei Yanagisawa, Yosuke Ogata, Shun Okuwaki, Masaki Tatsumura

**Affiliations:** 1 Orthopaedic Surgery, University of Tsukuba, Tsukuba, JPN; 2 Orthopaedic Surgery and Sports Medicine, Tsukuba University Hospital Mito Clinical Education and Training Center, Mito Kyodo General Hospital, Mito, JPN

**Keywords:** l5 spinal nerve injury, perioperative complication, s2 alar-iliac screw, sacroiliac joint, surgical case report

## Abstract

Although neurovascular structures, including the superior gluteal artery, sciatic nerve, obturator nerve, internal iliac vein and artery, and lumbosacral plexus, are at risk when S2 alar-iliac (S2AI) screws are used, no cases of nerve injuries have been reported. An 84-year-old man was referred to our institute with persistent left sciatica for seven months after undergoing salvage surgery using S2AI screws for deep surgical site infection from a previous posterior interbody fusion surgery at L5-S1. Based on the radiographic and diagnostic selective nerve root block findings, a diagnosis of left L5 radiculopathy was suspected due to the left S2AI screw being caudally misplaced and severely protruding into the pelvic cavity. The patient underwent surgical replacement of the left S2AI screw. The patient was discharged eight days postoperatively, with a resolution of the left leg pain. At the three-month follow-up, no recurrent pain was reported.

To the best of our knowledge, this is the first reported case of a misplaced S2AI screw causing L5 spinal nerve injury. If the screw is inserted caudally and deviates into the pelvic cavity, in front of the sacroiliac joint, the L5 nerve running in this region may be damaged.

## Introduction

The S2 alar-iliac (S2AI) screw, initially described by Sponseller and Kebaish as a new spinopelvic fixation technique, was reported in 2009 by Chang et al. [1] and O’Brien et al. [2]. The S2AI screw is a modification of the traditional iliac fixation technique that uses the space between the posterior foramen of S1 and S2 as an insertion point for fixing the sacrum to the ilium, which traverses three cortices, providing a strong purchase [1,2]. This technique reduces tissue morbidity by limiting the incision size and reducing paraspinal muscle dissection. Furthermore, connection to the rod construct and the S2AI screw usually requires no side connector. S2AI screws have no statistical differences in stiffness and load-to-failure compared with traditional iliac screws [3]. However, lower complication rates have been reported with S2AI screws, including mechanical failure, revision, loosening, and wound complications due to hardware prominence [4,5]. Therefore, S2AI screws are widely used as alternatives to traditional iliac screws for spinopelvic fixation.

Neurovascular structures, including the superior gluteal artery, sciatic nerve, obturator nerve, internal iliac vein and artery, and lumbosacral plexus, are at risk with S2AI screws [6]. Although the accuracy of S2AI screws is reportedly very high, even in large-scale analyses (>100 screws) [7-9], sometimes the screws were misplaced by breaching the cortex of the sacrum or ilium, with a breach rate of 5% for freehand [7], 1.7% for O-arm navigation [8], and 2.2% for robotic guidance [9]. However, no individual cases of neurovascular injury with S2AI screws have been reported. To the best of our knowledge, we present the first case of a misplaced S2AI screw causing L5 spinal nerve injury.

## Case presentation

An 84-year-old man visited our institute with persistent left sciatica for seven months after undergoing third surgery at another hospital. He had no comorbidities. He underwent a posterior lumbar interbody fusion at L4-L5 as the primary surgery for degenerative spondylolisthesis one year and seven months prior at another hospital. Although his symptoms improved postoperatively, he underwent an additional posterior interbody fusion at L5-S1 as the secondary surgery for adjacent segment disease nine months prior. Unfortunately, a delayed deep surgical site infection (SSI) occurred seven months prior, and salvage treatment was performed as the third surgery, including removal of the infected interbody cage at L5-S1 and posterior extended fixation to the pelvis using S2AI screws. Immediately after the third surgery, the patient developed new-onset neurological pain on the lateral aspect of the left lower leg. Despite conservative treatment with oral mirogabalin 10 mg/day, the symptoms did not improve.

During the first visit to our institute, the patient complained of severe pain and numbness radiating from the left buttock to the lateral aspect of the lower leg on standing up, which worsened on walking. The visual analog scale (VAS) score for leg pain was 80/100 mm. The straight leg-raising test was limited to 70° on the left side, without lower limb muscle weakness. Hypalgesia and hypesthesia were not observed. The blood tests showed no inflammatory response: white blood cell count, 3,400/μL (reference values: 3,100-8,400/μL); C-reactive protein, 0.04 mg/dL (reference values: ≤0.3 mg/dL).

**Figure 1:**
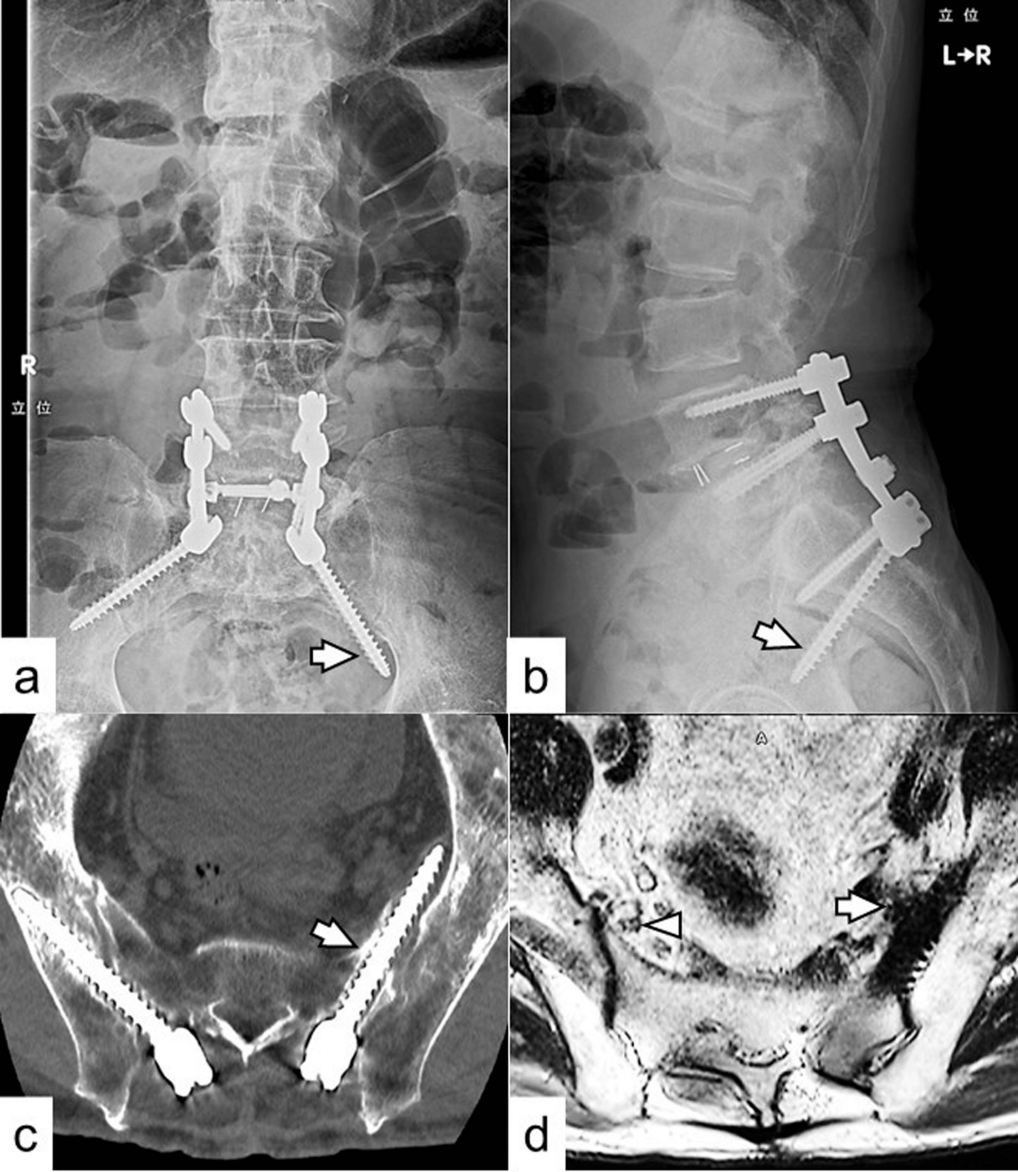
Imaging findings during the patient’s first visit to our institute. Anteroposterior (a) and lateral (b) views of the lumbosacral spinal plain radiography reveal a caudally misplaced S2AI screw on the left side (arrow). (c) CT shows the left S2AI screw severely protruding into the pelvic cavity and breaching the anterior cortex of the sacrum (arrow). (d) T2-weighted MRI reveals that the left L5 spinal nerve is markedly displaced anteriorly by the misplaced S2AI screw (arrow) compared to the right L5 spinal nerve, which is located just medial to the inferior sacroiliac joint (arrowhead). S2AI, S2 alar-iliac; CT, computed tomography; MRI, magnetic resonance imaging

Lumbar spine radiography revealed a caudally misplaced left S2AI screw (Figures 1a-1b). Computed tomography (CT) showed that the left S2AI screw was protruding severely into the pelvic cavity and breached the anterior cortex of the sacrum (Figure 1c). The screw diameter was 8.5 mm. T2-weighted magnetic resonance imaging (MRI) revealed marked anterior displacement of the left L5 spinal nerve by the misplaced S2AI screw, compared with the right L5 spinal nerve, which was located just medial to the inferior sacroiliac joint (Figure 1d).

**Figure 2:**
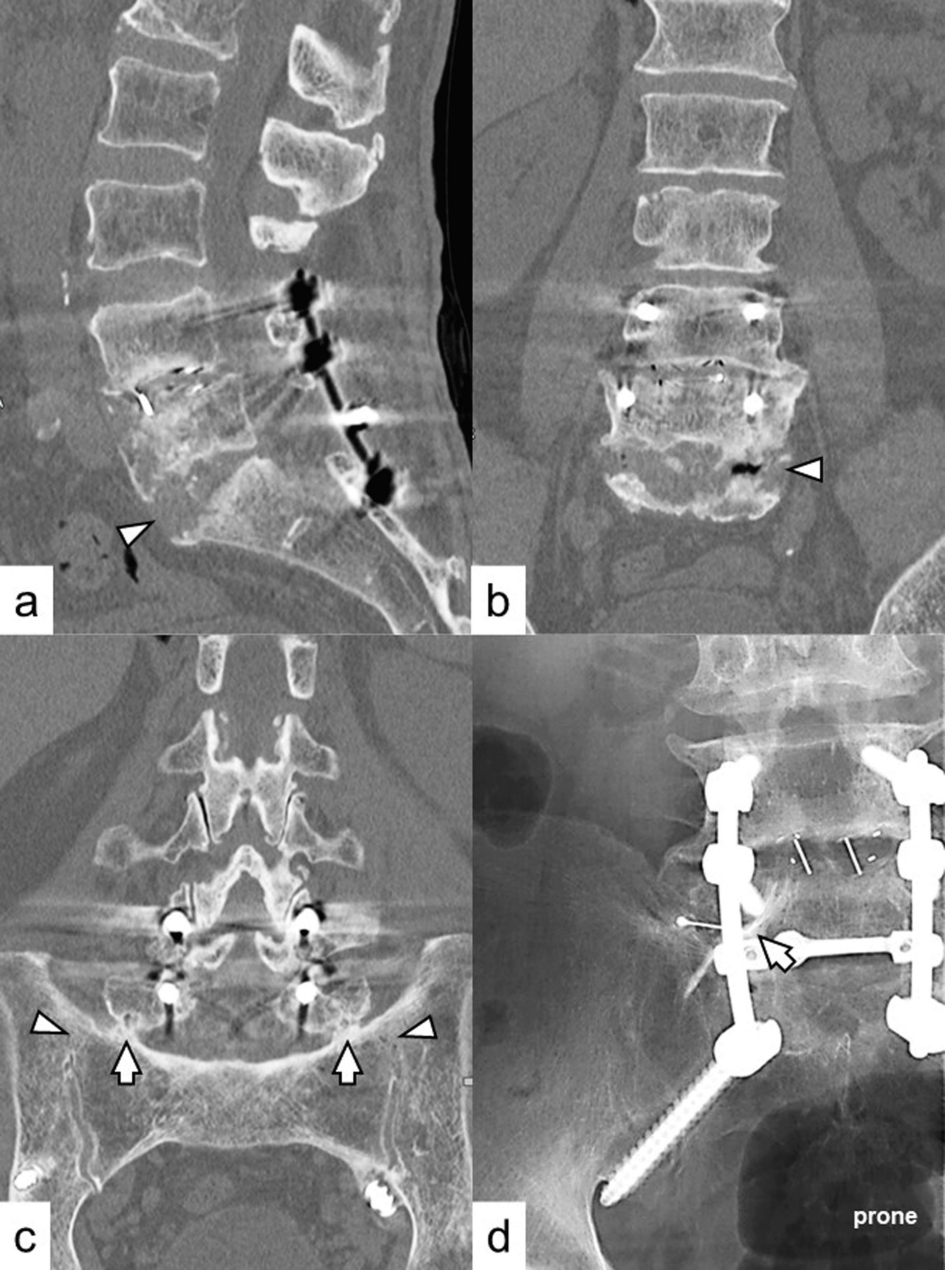
Imaging findings before surgery at our institute. According to the sagittal (a) and coronal (b) CT scans, bony fusion is not yet achieved at the L5-S1 intervertebral space, where the infected interbody cage has been removed (arrowheads). (c) Bony fusion between the transverse process of L5 and sacral alar (arrows) is evident bilaterally on CT. Furthermore, the bilateral sacroiliac joints have fused spontaneously (arrowheads). (d) The contrast medium in the left L5 diagnostic selective radiculography indicates that the left L5 nerve root is not compressed in the L5-S1 foraminal area (arrow), and no foraminal stenosis at L5-S1 is detected. CT, computed tomography

Although bony fusion of the L5-S1 intervertebral space, where the infected interbody cage was removed, had not yet been achieved (Figures 2a-2b), bony fusion between the transverse process of L5 and the sacral alar was evident bilaterally on CT (Figure 2c). Based on these findings, the deep SSI was considered well-controlled, and healing showed progress. To identify the responsible spinal level, we performed a left L5 diagnostic selective nerve root block following radiculography. When the needle reached the left L5 nerve root, the patient experienced radicular pain in the same area on the lateral aspect of the left lower leg that the patient had complained of. Subsequently, a clear analgesic effect was observed immediately after the block. However, the analgesic effect lasted only a few hours. The contrast medium indicated that the left L5 nerve root was not compressed at the L5-S1 foraminal area (Figure 2d); no foraminal stenosis at L5-S1 was detected. Furthermore, sacroiliac joint disorder, often presenting symptoms such as radiculopathy [10], was excluded because of the spontaneous fusion of the sacroiliac joint bilaterally (Figure 2c). Therefore, a diagnosis of left L5 radiculopathy due to a misplaced S2AI screw in front of the inferior sacroiliac joint was made.

**Figure 3:**
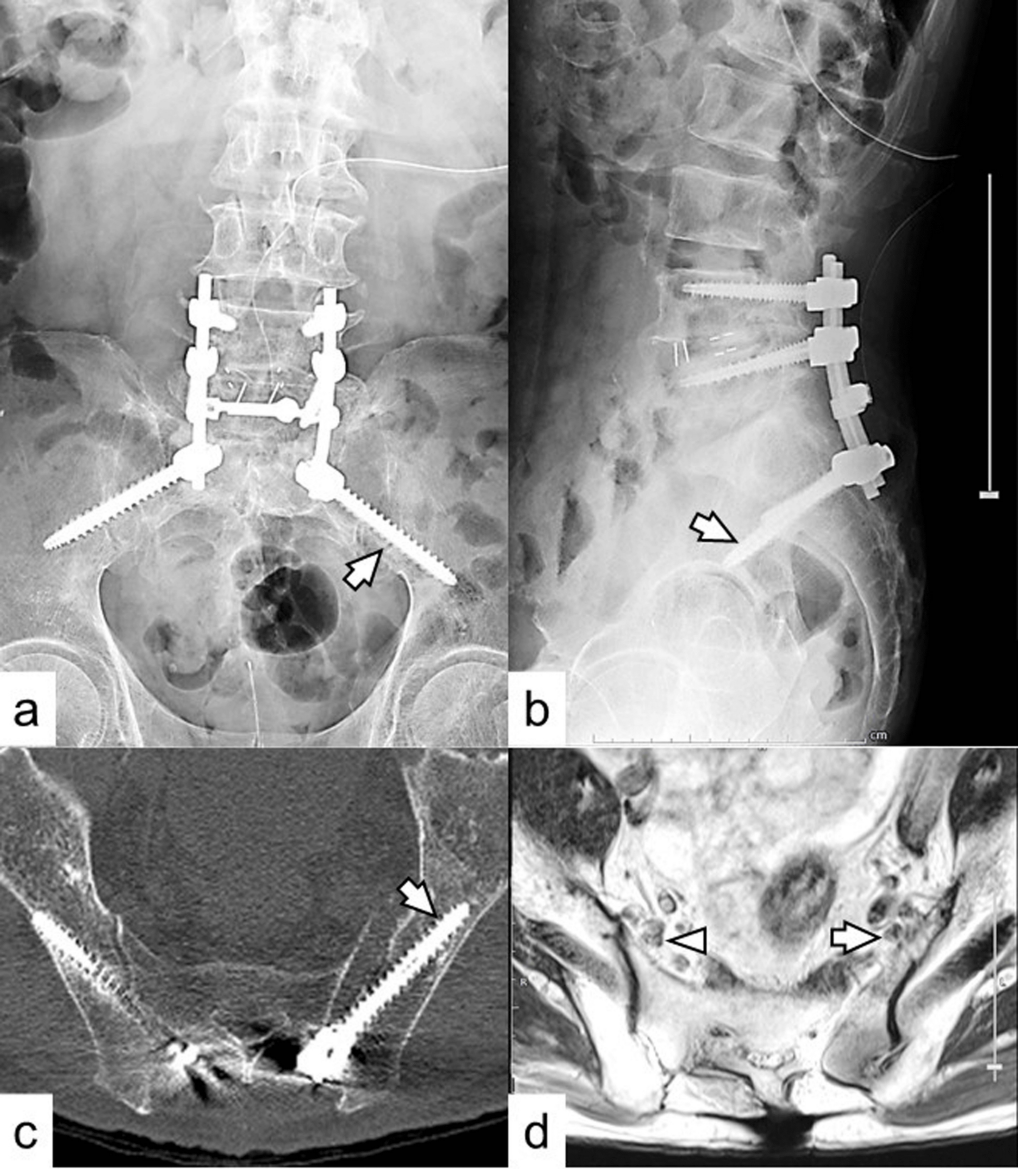
Postoperative images. Anteroposterior (a) and lateral (b) views of the plain radiography and CT (c) reveal the replaced left S2AI screw in the correct trajectory (arrows). The right S2AI screw was also replaced with a larger diameter screw because of slight loosening. (d) T2-weighted MRI revealed that the left L5 spinal nerve had returned to its original location (arrow) compared to the preoperative image (Figure 1d), and there was no difference in the position of the right L5 spinal nerve (arrowhead). S2AI, S2 alar-iliac; CT, computed tomography; MRI, magnetic resonance imaging

The fourth surgery was performed at our institute for S2AI screw replacement. During intraoperative neurological function monitoring, free-run waveforms that indicate direct nerve dysfunction were not detected on electromyography. Intraoperative screw-triggered electromyography, which also shows direct nerve dysfunction, yielded negative results. The left S2AI screw was replaced with the correct trajectory using fluoroscopic imaging (Figures 3a-3c). The right S2AI screw was also replaced with a larger diameter screw because of slight loosening. The left leg pain markedly improved to a VAS score of 20 mm immediately after the surgery. The postoperative course was uneventful, and the patient was discharged after eight days. T2-weighted MRI revealed that the left L5 spinal nerve had returned to its original location (Figure 3d). Although the patient complained of numbness, no recurrent pain was reported three months postoperatively.

## Discussion

Although reporting and publication biases have probably led to no previous reports, to the best of our knowledge, this is the first case of a misplaced S2AI screw causing L5 radiculopathy. The entry point of the S2AI screw is located just lateral to the midpoint between the S1 and S2 posterior foramen, and it should be in line with the proximal S1 anchors. The pedicle probe is directed toward the anterior inferior iliac spine (AIIS) by aiming at a point cephalad to the posterior distal edge of the posterior superior iliac spine and perpendicular to the lateral sacral crest, avoiding the sciatic notch inferiorly [7]. Due to anterior misplacement, the probe often slips on the hard sacroiliac joint surface of the ilium and breaches the sacroiliac joint anteriorly [8]. However, it does not usually cause neurovascular injury and is asymptomatic [7-9].

Anatomically, the L5 spinal nerve exits the intervertebral foramen, combines with the L4 spinal nerve (lumbosacral trunk), passes very close inside the inferior part of the sacroiliac joint, and combines with the S1 spinal nerve at the lumbosacral plexus of the lesser pelvis [11-13]. Therefore, if the screw trajectory is toward the AIIS, the spinal nerve will not be damaged in case of anterior misplacement of the S2AI screw through the sacroiliac joint because the spinal nerve passes more medially from the sacroiliac joint. Hence, the anterior misplacement of S2AI screws is usually asymptomatic. In contrast, the misplaced S2AI screw in this case was directed caudally and protruded into the pelvic cavity, breaching the anterior cortex of the sacrum at the inferior part of the sacroiliac joint, where the spinal nerve passed. Hence, the misplaced S2AI screw in this case caused radiculopathy. The combining level of the L4 and L5 spinal nerves may be above, on, or below the anterior-most part of the sacroiliac joint [14]. The diagnostic selective nerve root block showed that radiculopathy in this case was isolated at the L5 spinal nerve. Therefore, the combining level of the L4 and L5 nerves was considered more inferiorly than that of the L5 spinal nerve compressed by the misplaced S2AI screw.

Regarding symptom severity, the patient complained of severe radiculopathy, with a VAS score of 80/100 mm. Preoperative MRI showed that the L5 spinal nerve had been displaced anteriorly by the misplaced S2AI screw, but it was still visible. Moreover, intraoperative neurological function monitoring did not show a free-run waveform, and the screw-triggered electromyography was also negative. Therefore, we believe that the left L5 spinal nerve was severely compressed and displaced by the misplaced S2AI screw, rather than being directly damaged. No significant movement or displacement of the L5 spinal nerve (<1 mm) in the pelvic cavity during flexion/extension of the hip and lower lumbar spine was reported [15]. Thus, the L5 spinal nerve had poor mobility within the pelvic cavity. This could explain why a simple nerve compression and displacement within the pelvic cavity caused such severe symptoms.

## Conclusions

If the S2AI screw is inserted caudally and deviates into the pelvic cavity in front of the sacroiliac joint, it may compress or damage the L5 nerve, which runs in close proximity to it. Spinal nerve compression or injury should be considered when a patient experiences radicular leg pain as a new symptom after the insertion of S2AI screws.
